# Supplementary material for: How air pollution affects corporate total factor productivity?
Source: PLoS One. 2024 May 24;19(5):e0304079. doi: 10.1371/journal.pone.0304079 (PMC11125488; doi:10.1371/journal.pone.0304079)
Supplement: Attachment — Submitted filename: Response to Reviewers.docx [file pone.0304079.s002.docx]

Dear Professor Shen

Thank you very much to the editor and reviewers for their valuable revision suggestions, which are of great significance to our paper. According to the questions from the teachers, the response is as follows:

Question: Please include this amended Role of Funder statement in your cover letter; we will change the online submission form on your behalf ?

Answer: The funders had no role in study design, data collection and analysis, decision to publish, or preparation of the manuscript. We have deleted relevant information in the Acknowledgement.

Question: Funding information should not appear in the Acknowledgments section or other areas of your manuscript. We will only publish funding information present in the Funding Statement section of the online submission form. Please remove any funding-related text from the manuscript and let us know how you would like to update your Funding Statement.

Answer: We have removed the funding-related text in the Acknowledgement section. Our amended statements as follows: This research was funded by [Top-notch Academic Programs Project of Jiangsu Higher Education Institutions (Provincial First-class Specialty Construction of Energy Economy in Jingjiang College of Jiangsu University)].

Question: Please ensure that you have an ORCID iD and that it is validated in Editorial Manager.

Answer: The corresponding author already has it (ID: 0009-0002-4234-8737).

Question: We note that [Figure 1] in your submission contain [map/satellite] images which may be copyrighted.

Answer: Thank you for your correction, We have removed the Figure 1.

Question: Have the authors made all data underlying the findings in their manuscript fully available?

Answer: We have supplemented the provided data, please refer to Table S1 for details.

Here are the responses to the two reviewer teachers.

Reviewer #1:

Question: The article has a poor analysis of the literature, and there is no research gap. The hypotheses were formulated based on what? Why was this methodology adopted? what does this mean? The lack of these assumptions precludes further analysis.

Answer: Thank you for your correction, we have rewritten the introduction and literature review sections, summarized the latest research progress, proposed innovations in this article, and proposed hypotheses based on existing research conclusions and gaps.

Reviewer #2:

Question1: Abstracts are excessively long. It fails to succinctly elucidate the purpose or gaps in the research.

Answer: Thank you for your correction. We have rewritten the abstract section, shortened the length, and clarified the purpose.

Question 2: The Introduction section is poorly designed. The gaps between this study and existing studies are not visible in the Introduction. Meanwhile, this section fails to give the research innovation.

Answer: Thank you for your correction. We have completely rewritten the introduction section, citing current research conclusions and pointing out the innovative points of this article.

Question 3: The Literature Review is weak in supporting the research hypotheses. Firstly, the Literature Review of this study fails to support the research hypotheses well and needs to be further strengthened. Secondly, the formulation of research hypotheses needs not only the support of authoritative literature but also the support of classical theories.

Answer: In response to your question, we have made modifications by summarizing the latest existing literature as the basis for the research design of this article, and proposing a new research path for this article.

Question 4: Does the interactive effect of research hypothesis 2 and research hypothesis 3 exist? For example, the government's attention impacts investor sentiment. Therefore, this section needs to be redesigned.

Answer: Thank you for your correction. We have redesigned this part of the hypothesis and analyzed the two paths separately to avoid any interaction. At the same time, this article has conducted endogeneity and robustness tests on the empirical results to ensure their rationality.

Question 5: The "Ethics Statement" on page 7 does not seem appropriate here.

Answer: Thank you for your correction. We fully agree with your point of view and have deleted this section.

Question 6: Research data is too old. The author mentions in the limitations section, “Due to the availability of data, the data in this paper is only up to 2019.” Is this an insurmountable challenge? If possible, please update the research data and reiterate the limitations.

Answer: Thank you for your correction. Due to a significant lack of data on listed companies in later years, in order to ensure a sufficient sample size, we have chosen the current year for analysis. Based on your suggestion, we have made modifications to the limitations. Thank you again.

Question 7: This study fails to provide descriptive statistics for all variables, which weakens the scientific validity of the empirical research.

Answer: Thank you for your correction. We fully agree and have added a descriptive statistics section, as shown in Table 2.

Question 8: The Discussion section is extremely poor. The present Discussion simply analyses the empirical results and does not compare them with existing studies to clarify how this study contributes to, adds to, or differs from existing studies. It is more a test of heterogeneity than a discussion.

Answer: Thank you for your correction. We have added a new discussion section and changed the previous discussion section to heterogeneity analysis.

Question 9: Policy recommendations need to be more specific, nuanced, and feasible. In addition, limitations and future research need to be more detailed.

Answer: Thank you for your correction. We have rewritten the policy recommendations section to make it more targeted. Meanwhile, the limitations have also been rewritten.

Question 10: English level needs to be improved. It has grammatical errors and presentation errors. In addition, this study needs to pay attention to the use of long sentences, acronyms, and proper nouns.

Answer: Thank you for your correction. We have improved our English.

Best wishes
